# Gestational age modifies the association between exposure to fine particles and fetal death: findings from a nationwide epidemiological study in the contiguous United States

**DOI:** 10.1186/s12940-023-01016-4

**Published:** 2023-09-14

**Authors:** Mingkun Tong, Weiwei Lin, Hengyi Liu, Jicheng Gong, Junfeng (Jim) Zhang, Tao Xue

**Affiliations:** 1 Institute of Reproductive and Child Health, National Health Commission Key Laboratory of Reproductive Health and Department of Epidemiology and Biostatistics, Ministry of Education Key Laboratory of Epidemiology of Major Diseases (PKU), School of Public Health , Peking University Health Science Center, Beijing, China; 2 Department of Occupational and Environmental Health, School of Public Health, Sun Yat-Sen University, Guangzhou, China; 3 State Environmental Protection Key Laboratory of Atmospheric Exposure and Health Risk Management and Center for Environment and Health, Peking University, Beijing, China; 4 Global Health Research Center, Duke Kunshan University, Kunshan, China; 5 Nicholas School of the Environment and Duke Global Health Institute, Duke University, Durham, USA; 6 Advanced Institute of Information Technology, Peking University, Hangzhou, Zhejiang, China

**Keywords:** Fetal death, PM_2.5_, Prenatal exposure, Effect modification, Gestational age

## Abstract

**Backgrounds:**

The vulnerability of fetuses differs at different developmental stages, in response to environmental stressors such as fine particulate matter (PM_2.5_), a ubiquitous air pollutant. Whether gestational age (GA) modifies the association between prenatal fine particulate matter (PM_2.5_) exposure and fetal death remains unclear.

**Methods:**

We selected approximately 47.8 million eligible United States (US) livebirth and fetal death (defined as a termination at a GA of 20–43 weeks) records from 1989 to 2004. For each record, we took the level of prenatal exposure to PM_2.5_ as the average concentration in the mother’s residential county during the entire gestational period, or a specific trimester (i.e., GA-specific exposure), according to well-established estimates of monthly levels across the contiguous US. First, we evaluated the associations between PM_2.5_ exposure and fetal death at a specific GA (i.e., GA-specific outcome) using five different logit models (unadjusted, covariate-adjusted, propensity-score, double robust, and diagnostic-score models). Double robust model was selected as the main model due to its advantages in causal inference. Then, we conducted meta-analyses to pool the estimated GA-specific associations, and explored how the pooled estimates varied with GA.

**Results:**

According to the meta-analysis, all models suggested gestational PM_2.5_ exposure was associated with fetal death. However, there was slight heterogeneity in the estimated effects, as different models revealed a range of 3.6–10.7% increase in the odds of fetal death per 5-µg/m^3^ increment of PM_2.5_. Each 5-µg/m^3^ increase in PM_2.5_ exposure during the entire gestation period significantly increased the odds of fetal death, by 8.1% (95% confidence interval [CI]: 5.1–11.2%). In terms of GA-specific outcomes, the odds of fetal death at a GA of 20–27, 28–36, or ≥ 37 weeks increased by 11.0% (5.9–16.4%), 5.2% (0.4–10.1%), and 8.3% (2.5–14.5%), respectively. In terms of GA-specific exposure, the odds of fetal death increased by 6.0% (3.9–8.2%), 4.1% (3.9–8.2%), and 4.3% (0.5–8.2%) with 5-µg/m^3^ increases in PM_2.5_ exposure during the first, second, and third trimester, respectively. The association had the largest effect size (odds ratio = 1.098, 95% CI: 1.061–1.137) between PM_2.5_ exposure during early gestation (i.e., first trimester) and early fetal death (i.e., 20–27 weeks).

**Conclusions:**

Prenatal exposure to PM_2.5_ was significantly associated with an increased risk of fetal death. The association was varied by gestational-age-specific exposures or outcomes, suggesting gestation age as a potential modifier on the effect of PM_2.5_. The fetus was most vulnerable during the early stage of development to death associated with PM_2.5_ exposure.

**Supplementary Information:**

The online version contains supplementary material available at 10.1186/s12940-023-01016-4.

## Introduction

Fetal death (or stillbirth) is an important but previously neglected public health issue. For instance, reducing fetal death was not included as one of the Millennium Development Goals. Despite the scant global attention on this issue, fetal death is associated with substantial direct (e.g., medical expenditure), indirect (e.g., unemployment), and intangible (e.g., mental stress caused by anxiety or grief) costs to women and their families, governments, and even entire societies [1]. In 2019, the estimated global rate of stillbirth during the third trimester was 13.9 per 1,000 total births (90% uncertainty range: 13.5–15.4), and nearly 83.6% of stillbirths occurred in low-income and lower-middle income countries [2]. Preventing fetal death is not only beneficial with respect to reproductive health, but also promotes social equity. Therefore, it is necessary to identify the modifiable risk factors.

An association has been reported between prenatal exposure to ambient fine particulate matter (PM_2.5_) and fetal death in North America [3], Europe [4], East Asia [5], South Asia [6], and Africa [7]. Although gestational PM_2.5_ exposure generally increases the risk of fetal death, the strength of the effect appears to be modulated by gestational age (GA), for a number of reasons. First, the vulnerability of embryos varies by developmental stage, so the baseline risk of fetal death changes with GA. This baseline risk, which depends on the presence of factors that may interact with the effects of air pollution, can influence the strength of the association between PM_2.5_ and fetal death. For instance, a previous study reported that the odds ratio (OR) of stillbirth (death between 20 and 44 gestational weeks) was 1.06 (95% confidence interval [CI]: 1.01–1.11) per 7.23-µg/m^3^ increase in PM_2.5_, while the OR of stillbirth before and after 28 gestational weeks was 1.05 (95% CI: 0.99–1.12) and 1.09 (95% CI: 1.01–1.17), respectively [8]. Another study divided stillbirths into several GA strata (i.e., 23–26, 27–30, 31–36, and 37–42 weeks); the corresponding relative risks (RRs) were 1.12 (95% CI: 0.57–2.20), 1.53 (95% CI: 0.66–3.52), 0.71 (95% CI: 0.40–1.28), and 0.84 (95% CI: 0.44–1.59) per interquartile range (IQR) increase in PM_2.5_, respectively [9]. These studies suggest that GA can modify the effect of PM_2.5_ on fetal survival. However, the statistical power in previous studies was insufficient because of limited sample sizes after stratification by GA.

Additionally, developmental stage determines the susceptibility of an embryo to adverse environments. Therefore, PM_2.5_ concentrations have been found to have differential effects according to GA. Most previous studies assessed exposure to PM_2.5_ concentrations averaged over the entire gestational period, or a specific trimester. For instance, two recent meta-analyses reported that each 10-µg/m^3^ increase in PM_2.5_ exposure over an entire pregnancy was associated with a 15% (95% CI: 7–25%) or 10% (95% CI: 7–13%) increase in the risk of stillbirth. Both studies found that the association was statistically significant for third-trimester PM_2.5_ exposure, but not for first- or second-trimester exposure [10, 11]. These results suggest that the adverse effects of PM_2.5_ on fetal survival can vary according to GA. However, it is difficult to compare the findings of previous studies because the fetal death rates were analyzed for different GA-specific exposure times. For instance, fetal deaths that occurred at an early GA (also known as an early stillbirth) could not be included in the analysis on the third-trimester exposure. In other words, to fully understand how GA modifies the association between PM_2.5_ and fetal death, GA-specific exposure and outcomes should be analyzed simultaneously.

Understanding how GA modifies the association between fetal death and PM_2.5_ has important consequences for public health. Usually, population exposure to PM_2.5_ is universal and prolonged, such that regular public interventions rely on long-term emission control measures, such as clean air action. If there is a specific time window during which per-unit exposure poses a particularly significant health risk, this risk could be addressed via personalized interventions (e.g., providing air purifiers to pregnant women). Therefore, identifying the time window for critical exposure is important for guiding the implementation of additional protections for developing fetuses.

In our previous study, we created a large population database by combining registration data on livebirths and fetal deaths across the contiguous United States (US) from 1989 to 2004, and utilized it to examine the effects of PM_2.5_ on fetal death. Here, we further evaluated the way in which GA modified this association to identify the most susceptible time window for PM_2.5_ exposure. Using a two-stage model, we estimated the associations for all possible pairs of GA-specific outcomes and GA-specific exposure times.

## Methods

### Study population

Our study participants were extracted from the publicly available datasets of the US National Center for Health Statistics, which collects birth and fetal death certificates (https://www.cdc.gov/nchs/data_access/vitalstatsonline.htm). Fetal death was defined as death prior to the complete expulsion or extraction from the mother of a product of human conception, irrespective of the duration of pregnancy, except for induced termination. Previous studies included stillbirth occurring from 20 to 42 or 44 weeks of gestation [12, 13]. The period of gestation is the number of completed weeks elapsed between the first day of the last menstrual period (LMP) and the date of delivery. According to the recommendation from US Centers for Disease Control and Prevention and the distribution of GA in our dataset, we included records in which GA was between 20 and 43 gestational weeks. For GA-specific outcomes, we defined early, late, and term fetal deaths as those occurring at a GA of 20–27, 28–36, and 37–43 weeks, respectively [14]. Only records created before 2005 were geocoded to the county level, because geographic codes have not been provided publicly since 2005 for reasons of confidentiality. According to the U.S. Census Bureau, there are more than 3000 counties in US, and more than half of all residents live in just 143 big counties with a median population of 821,725 (926 people per square mile), the remaining small counties with a median population of 23,999 host less than half of the population (48 people per square mile). To maximize the number of records in the dataset, we imputed missing GA data when the birth date and LMP date were available. We excluded records with unknown GA after imputation (49,584 fetal deaths and 3,344,409 livebirths) or insufficient information, as described previously [15]. Ultimately, data for nearly 47.8 million fetuses between 1989 and 2004, from 527 counties across the contiguous US, were included in our analyses.

### Environmental variables

We estimated monthly PM_2.5_ levels at a high spatial resolution of 0.01° × 0.01° with a well-validated chemical transport model using satellite remote-sensing measurements and ground-based monitoring observations across North America from 1989 to 2004. Residential addresses were not available; we could only access county-level information about the locations of the pregnant mothers. First, we calculated county-level averages based on administrative boundaries. Then, each birth and fetal death record was matched with the environmental exposure data of the county of residence. As an indicator of overall exposure during pregnancy, we averaged all monthly PM_2.5_ values from the month of the LMP to the birth month for each eligible record. The average PM_2.5_ concentrations between the 1^st^ and 3^rd^ months and 4^th^ and 7^th^ months, and after the 7^th^ month (truncated at the corresponding month of last gestational age), were calculated to determine the first-, second-, and third-trimester exposure, respectively.

We also obtained monthly data on temperature, relative humidity, and wind speed from the North American Regional Reanalysis (NARR) project to control for the confounding effects of these climate variables. The NARR provides meteorological data for North America with a grid size of 32 km × 32 km; the data are freely available from the website of the NOAA-ESRL Physical Sciences Laboratory, Boulder, Colorado (https://psl.noaa.gov/data/gridded/data.narr.html). The assessment process was the same for the climate and PM_2.5_ exposure data.

### Covariates

The birth and fetal death certificates included maternal, paternal, and fetal information, which were transformed into categorical variables and adjusted for potential confounders including maternal age (≤ 15, 16–20, 21–25, 26–30, 31–35, 36–40, 41–45, or > 45 years), maternal education (≤ 8, 9–12, 13–16, or > 16 years), maternal and paternal ethnicity (White, African American, Chinese, American Indian/Alaskan Native, Japanese, Hawaiian, or other), marital status (yes or no), prenatal care attendance (yes or no), whether the infant was born in a hospital (yes or no), previous history of pregnancy (yes or no), plurality (one, two, or more than three fetuses), previous history of at least one livebirth (yes or no), history of abnormal terminations (none, one, two or ≥ three), maternal tobacco usage (yes or no), maternal alcohol usage (yes or no), chronic diabetes (yes or no), hypertension (yes or no), gestational weight gain (≤ 15, 16–20, 21–25, 26–30, 31–35, 36–40, 41–45, or > 45 pounds), and fetal sex (female or male).

### Statistical analysis

We used a two-stage analysis approach. In the first stage, we conducted a series of regressions to evaluate the associations between GA-nonspecific PM_2.5_ exposure (i.e., exposure during the entire gestation period) and GA-specific PM_2.5_ exposure (i.e., exposure during a specific trimester) with GA-specific fetal death (i.e., fetal death at a specific GA). Second, we combined these GA-specific results via meta-analysis or meta-regression to explore GA-dependent patterns in the association between PM_2.5_ exposure and fetal death.

After stratifying the data according to gestational week, we used a logistic regression model with fetal deaths taken as cases and all livebirths as controls to estimate the GA-specific association between PM_2.5_ exposure and fetal death for each stratum, based on the OR of fetal death per 5-µg/m^3^ increment in gestational PM_2.5_ exposure. Without adjustment, this type of analysis is classified as an unmatched case-control study. As such an analysis can be easily confounded, we fitted four other models, including causal inference models. Below, we describe the four models in detail.

#### Covariate-adjusted logistic model

To employ this widely used method, we adjusted for a set of individual- and county-level covariates within each GA-stratum:

1 $$\mathrm{Logit}\left(y_i\right)={\beta x}_i+b{\boldsymbol z}_i+f_1({\mathrm T}_i)\;+f_2({\mathrm{RH}}_i)\;+f_3({\mathrm{WS}}_i)\;+\;{\gamma\boldsymbol s}_{\boldsymbol i}\;+\;{\kappa\boldsymbol t}_{\boldsymbol i}\;+\;{\lambda\boldsymbol m}_{\mathbf i}$$

where *i* indicates a given fetus, *y*_*i*_ denotes the binary outcome (1: fetal outcome; 0: normal delivery) for a specific fetus, *x*_*i*_ denotes the average PM_2.5_ concentration over the full gestation period for a specific fetus, **z**_*i*_ denotes the abovementioned individual-level covariates used for adjustment, and *f*_*1*_, *f*_*2*_, and *f*_*3*_ denote spline functions with three degrees of freedom for capturing the nonlinear effects of temperature (T), relative humidity (RH), and wind speed (WS), respectively. ***s***_*i*_ is a dummy variable representing the residential state, which we used to control for unmeasured confounders at the regional level, and ***t***_***i***_ and ***m***_***i***_ are dummy variables representing the birth year and birth season, to control for long-term trends and seasonality, respectively. *β, b, γ, κ*, and *λ* are regression coefficients. We used a code to denote missing values for all categorical variables except maternal marital status (0.79%). Because the marital status was likely unrecorded because of fetal death, the missing values were randomly imputed in such cases. This process to handle missing values was identical in other models listed followed.

#### Propensity score model

The propensity score (PS) is commonly used to make causal inferences when adjustment for confounders is needed. First, we used linear regression to estimate the conditional probability density function of the exposure (*x*_*i*_) given the independent variables shown in Eq. (1). Then, we created a pseudo-population using the PS weights, calculated as the inverse of the probability. For stabilization, the PS weights were trimmed using cut points at the 1^st^ percentile (low weights) and 99^th^ percentile (high weights). We fitted a weighted univariate logistic regression model to estimate the effect of PM_2.5_ exposure.

#### Double robust model

In the double robust model (DRM), PS weights and covariate adjustment are simultaneously applied. Thus, the effect estimation is robust to misapplication of one of these strategies [16]. We combined the multivariable logistic model of Eq. (1) with the above-mentioned PS weights to derive our main model before the data analysis.

#### Diagnostic score model

Similar to PSs, but with matching of outcomes to prevent confounding, diagnostic scores create comparable pseudo-populations of cases and controls. Therefore, this method represents a generalization of the matched case-control design. The diagnostic score weights were the inverse probabilities of an outcome given the measured confounders; the probabilities were derived from a logit regression identical to Eq. (1), except for the removal of exposure level (*x*_*i*_). We fitted a weighted univariate logistic regression model to estimate the effect of PM_2.5_ exposure.

In the second stage, we utilized a meta-analysis with random effects to pool GA-specific associations for the whole gestation period and each trimester. Heterogeneity in the GA-specific associations was evaluated using the I^2^ statistic. Considering the temporal auto-correlation in PM_2.5_ concentrations, we did not simultaneously regress the three trimester-specific exposures with fetal death, and estimated the effects of GA-specific exposure using separate two-stage models. Because of the high computational burden, only DRMs were developed in the first stage to estimate the effects of trimester-specific PM_2.5_ exposure. Note that not all GA-specific exposure-outcome pairs are theoretically possible. For instance, while the adverse effect of first-trimester PM_2.5_ exposure could be estimated for all fetal death subtypes, the influence of second-trimester exposure could only be evaluated for late and term fetal deaths (GA ≥ 28 weeks). Therefore, we derived pooled estimates for all possible GA-specific exposure-outcome pairs. Because of the high temporal resolution of the GA at fetal death data, we conducted a sensitivity analysis focusing on how GA modified the effect of PM_2.5_ exposure during the entire gestational period. We then performed a meta-regression of the GA-specific estimates against the spline expansion of GA in weeks (degrees of freedom = 4). All statistical analyses were performed using R packages. The PS and diagnostic scores were estimated using *ipw*, and the meta-analysis and meta-regressions were run in *meta*.

## Results

### Descriptive statistics

**Fig. 1:**
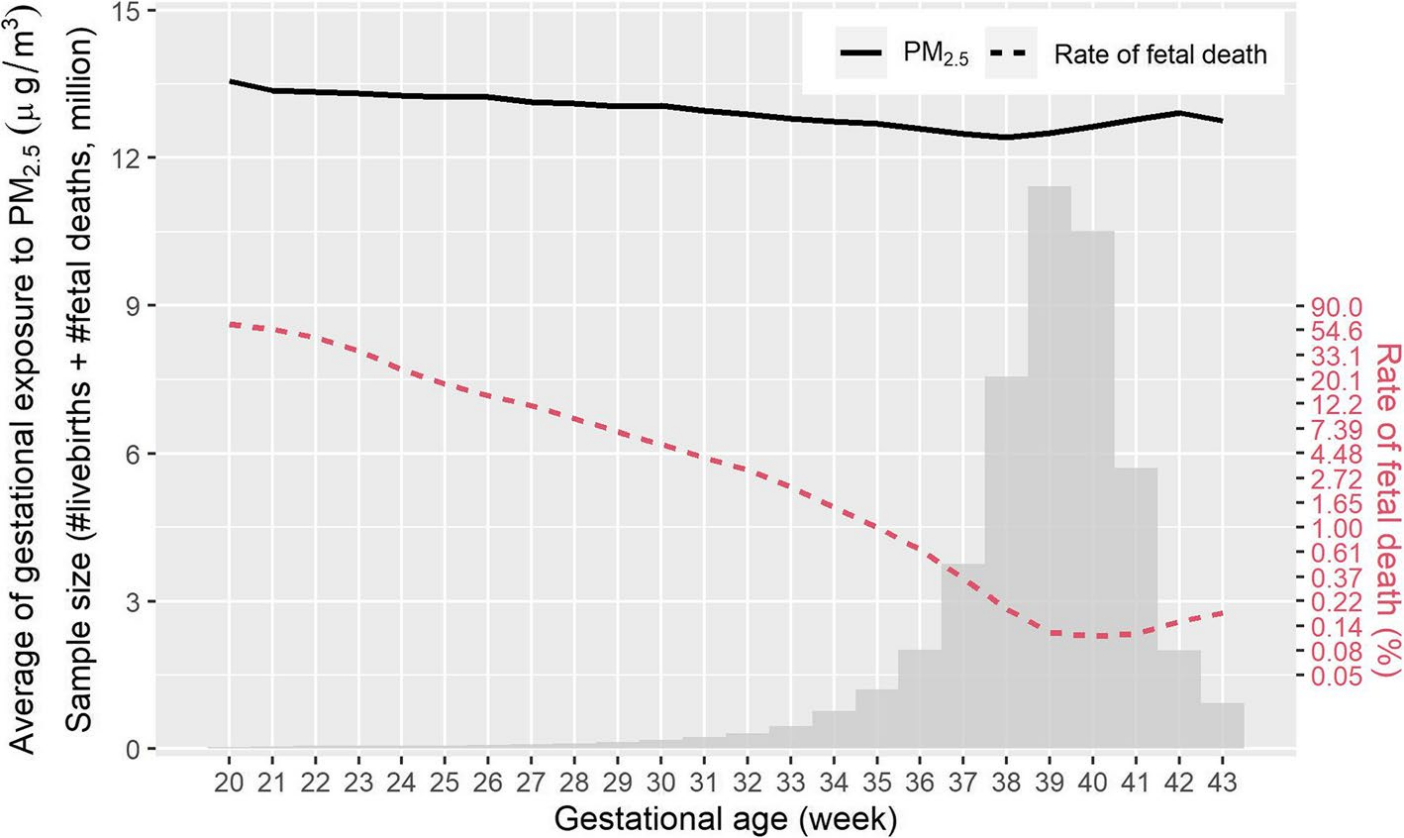
Fetal sample size, level of PM_2.5_ exposure during the entire gestation, and crude fetal death rate by subgroups of gestational weeks of fetal death

As shown in Table 1, of the 47.8 million cases analyzed in this study, 316,875 were recorded as fetal deaths (crude rate of 0.66%). Among the 527 counties, the median size of study population (including fetal deaths and livebirths) was 49,340 (P_2.5_ – P_97.5_: 3,170–388,769). Nearly 21.8 million of the fetuses had mothers (45.7%) who had received 9–12 years of education, and 31.8 million had mothers (66.6%) who were multipara. The mothers of 36.7 million (76.7%) and 7.8 million (16.3%) fetuses were white and African-American, respectively. The average age of the mothers was 27.3 (standard deviation [SD]: 6.1) years. The average exposure concentration of PM_2.5_ over the full gestational period was 12.6 (SD: 4.1) µg/m^3^, and the concentration in the fetal death group was higher than that in the livebirth group (13.2 ± 4.2 vs. 12.6 ± 4.1 µg/m^3^). The mean exposure concentration during the first, second, third trimester was 12.7 (SD: 4.5), 12.6 (SD: 4.3), and 12.5 (SD: 4.4) µg/m^3^, respectively. Detailed statistics regarding the population characteristics are shown in Table S1. Figure 1 shows the sample size, crude rate of fetal death, and PM_2.5_ exposure level by gestational week. The majority of the fetuses were born between 37 and 41 gestational weeks. The crude rate of fetal death varied with GA, showing a U-shaped trend whereby the lowest rate was at the 40^th^ gestational week. However, the average PM_2.5_ exposure during the entire gestational period was relatively stable, ranging from 12 to 14 µg/m^3^ among the GA-specific fetal death subgroups.

**Table 1:** Characteristics of study participants (from gestational 20^th^ week to 43^rd^ week). Other characteristics were summarized in Supplemental Table S1

Characteristics	Subgroup	N (%) or mean (standard deviation, interquartile range [IQR])
Total		47,845,444 (100.00)
Maternal age (years)		27.3 (6.1, 23.0–32.0)
Fetal outcome	Fetal death	316,875 (0.66)
	Livebirth	47,528,569 (99.34)
Years of maternal education	≤8	2,891,993 (6.04)
	9–12	21,854,907 (45.68)
	13–16	17,025,390 (35.58)
	17+	4,197,367 (8.77)
	Unknown	1,875,787 (3.92)
Previous pregnancy	Yes	31,844,671 (66.56)
	No	15,720,316 (32.86)
	Unknown	280,457 (0.59)
Maternal ethnicity	White	36,708,200 (76.72)
	African American	7,798,101 (16.30)
	Others	3,339,143 (6.98)
PM_2.5_ exposure (µg/m^3^)	Full gestational	12.6 (4.1, 9.7–15.5)
	First trimester	12.7 (4.5, 9.5–15.7)
	Second trimester	12.6 (4.3, 9.5–15.5)
	Third trimester	12.5 (4.4, 9.3–15.5)
Relative humidity (%)		70.1 (12.9, 68.0–78.1)
Temperature (°C)		14.1 (5.0, 10.2–17.5)
Wind speed (m/s)		3.5 (0.6, 3.0–3.8)

### Overall association between PM_2.5_ and fetal death estimated by different models

**Fig. 2:**
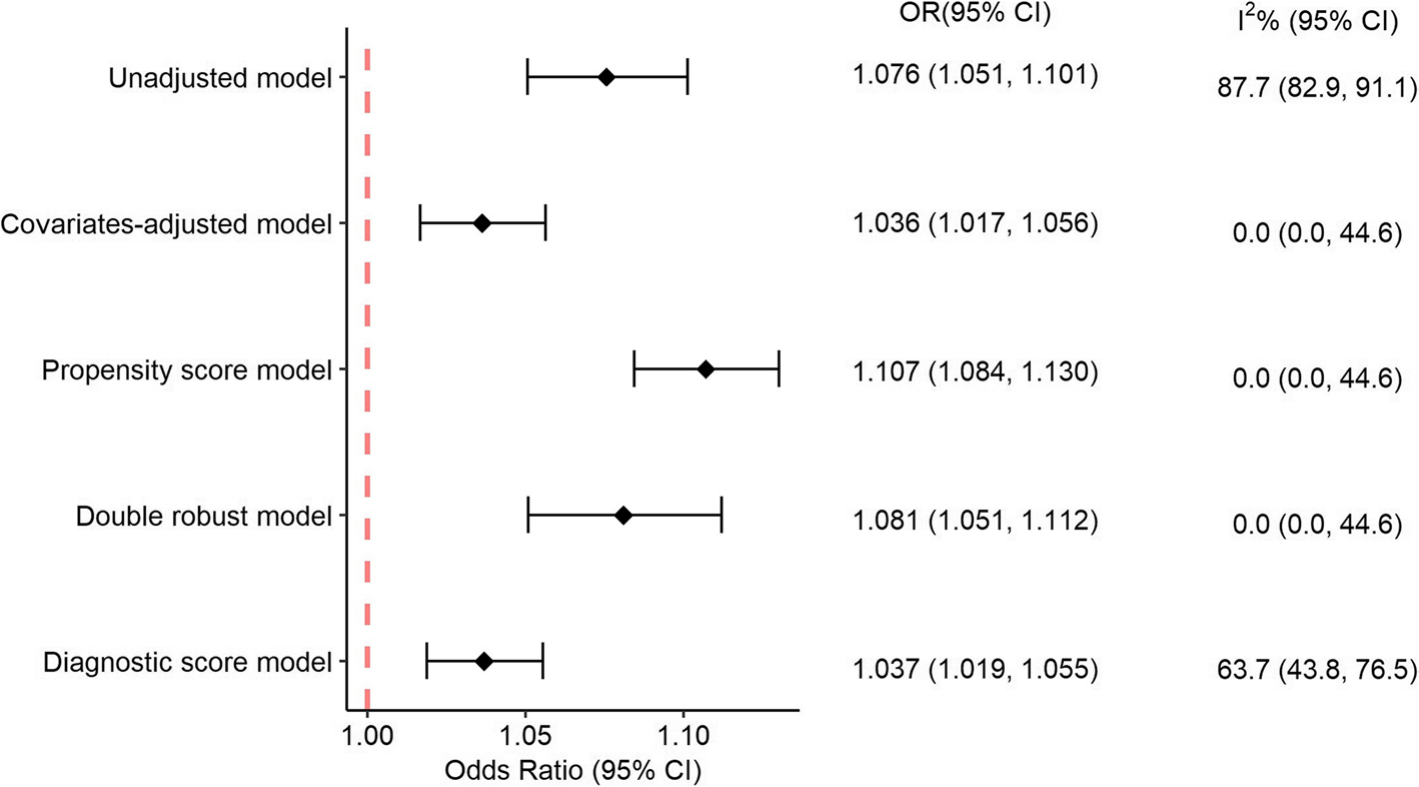
The overall association between fetal death and 5 µg/m^3^ increment in entire gestational PM_2.5_ exposure, estimated by different two-stage models. The pooled odds ratios (ORs) and their corresponding 95% confidence intervals (CIs) were derived from a random-effect meta-analysis of results from regressions stratified by gestational weeks. Heterogeneity among individual strata-specific estimates was evaluated using the I^2^ statistic

The pooled ORs from the different two-stage models consistently indicated a significant association between PM_2.5_ exposure and fetal death. However, the point estimates of the association varied according to the model settings (Fig. 2). According to our main model (i.e., the DRM), the OR of fetal death was 1.081 (95% CI: 1.051–1.112) per 5-µg/m^3^ increase in overall gestational PM_2.5_ exposure. The PS model, in which the population characteristics were balanced between groups with different exposure levels, yielded the strongest association (OR: 1.107; 95% CI: 1.084–1.130), followed by the main model, unadjusted model (OR: 1.076; 95% CI: 1.050–1.102), diagnostic score model (OR: 1.037; 95% CI: 1.019–1.056), and covariate-adjusted model (OR: 1.036; 95% CI: 1.017–1.056). Our meta-analysis showed that GA-specific results from the unadjusted and diagnostic models were heterogeneous, with estimated I^2^ statistics of 87.7% and 63.7%, respectively. No heterogeneity was found for the other three models, suggesting that their estimates were reliable. The following analyses are based on our main model (i.e., the DRM).

### GA-specific associations between PM_2.5_ and fetal death

The pooled ORs for associations between GA-specific PM_2.5_ exposure and GA-specific fetal death are shown in Table 2. First, with regard to GA-specific outcomes, the fetuses were categorized into early, late, and term fetal death groups. For each 5-µg/m^3^ increase in PM_2.5_ exposure during the entire pregnancy, the OR values for early, late, and term fetal death were estimated as 1.110 (95% CI: 1.059–1.164), 1.052 (95% CI: 1.004–1.101), and 1.083 (95% CI: 1.025–1.145), respectively. The differences between the estimated ORs of the GA-specific outcomes might have been due in part to the model settings (Fig. S1). However, the three homogenous models (i.e., the DRM, PS model, and covariate-adjusted model) yielded a similar trend: the largest OR corresponded to early fetal death and the smallest to late fetal death.

Second, the pooled ORs of all types of fetal death according to PM_2.5_ exposure during different trimesters varied slightly by GA-specific exposure. For each 5-µg/m^3^ increase in PM_2.5_ exposure during the first, second, and third trimester, the ORs were 1.060 (95% CI: 1.039–1.082), 1.041 (95% CI: 1.014–1.068), and 1.043 (95% CI: 1.005–1.082), respectively. Comparison of the ORs indicated that fetuses were susceptible to the toxic effects of PM_2.5_ during the early developmental stage (i.e., first trimester).

Last, for all possible GA-specific exposure-outcome pairs, except that including second-trimester exposure and term fetal death, the associations were statistically significant. The largest OR was for the association between first-trimester exposure and early fetal death (OR = 1.098, 95% CI: 1.061–1.137). This highlights the considerable impact of maternal PM_2.5_ exposure during early pregnancy on early fetal survival, suggesting that the first trimester is crucial for the prevention of fetal death.

**Table 2:** The pooled odds ratios (ORs) of fetal death for 5 µg/m^3^ increment in PM_2.5_ with 95% confidence intervals (CI), by specific subgroups of gestational age (GA). The GA-specific ORs are estimated by meta-analysis to pool results from double robust models together, for all possible GA-specific exposure-outcome pairs

PM_2.5_ exposure by GA	ORs of outcomes by GA (95% CI)			
	Early fetal death (20–27 weeks)	Late fetal death (28–36 weeks)	Term fetal death (37–43 weeks)	All fetal death (20–43 weeks)
First trimester (1^st^ − 3^rd^ month)	1.098 (1.061, 1.137)	1.037 (1.004, 1.071)	1.048 (1.008, 1.089)	1.060 (1.039, 1.082)
Second trimester (4^th^ − 7^th^ month)	–	1.059 (1.024, 1.095)	1.015 (0.974, 1.056)	1.041 (1.014, 1.068)
Third trimester (> 7^th^ month)	–	–	1.043 (1.005, 1.082)	1.043 (1.005, 1.082)
Entire gestation	1.110 (1.059, 1.164)	1.052 (1.004, 1.101)	1.083 (1.025, 1.145)	1.081 (1.051, 1.112)

### GA-dependent effect of entire-gestation exposure to PM_2.5_ on fetal death

**Fig. 3:**
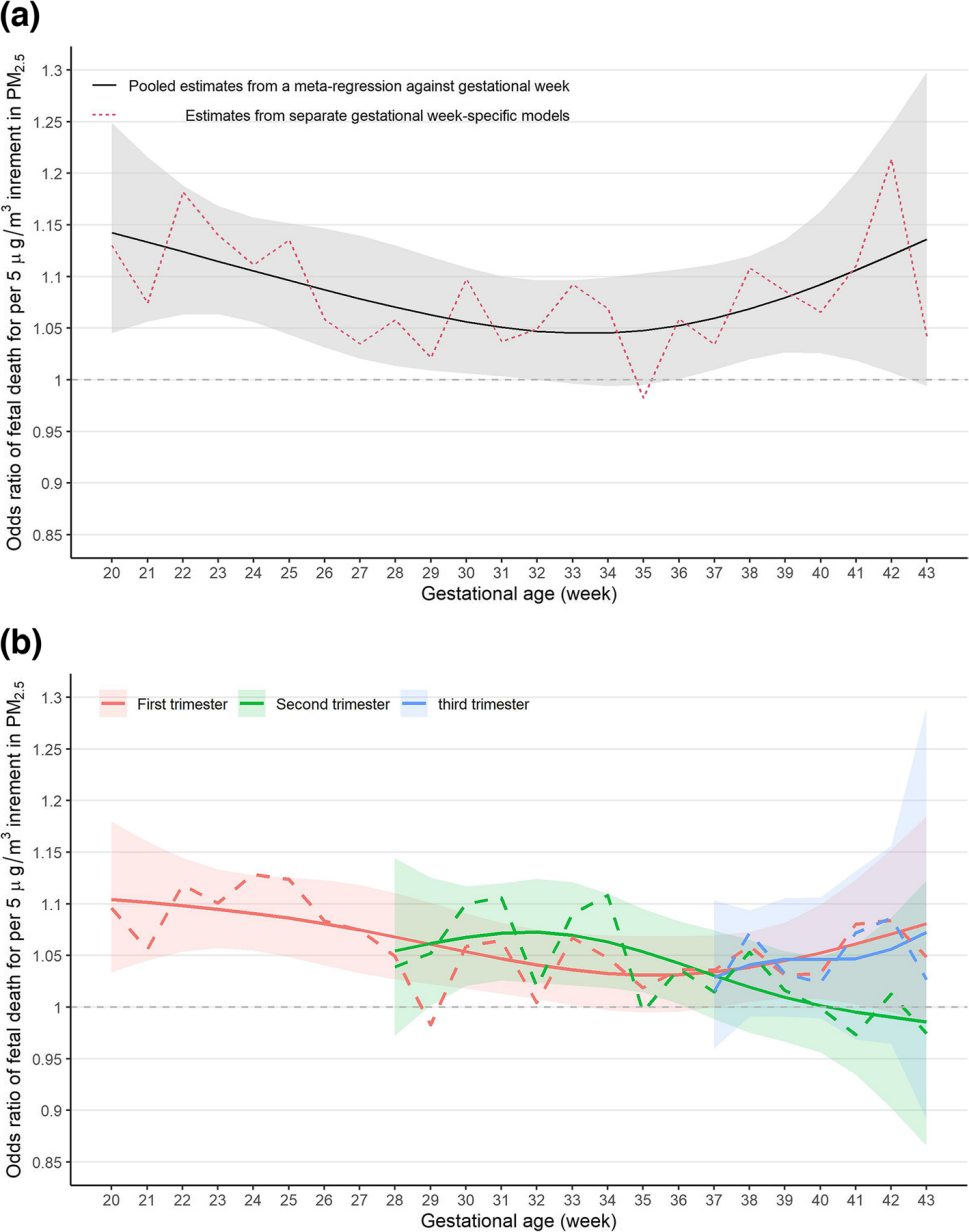
Variation in the estimated effect of PM_2.5_ exposure with different gestational week of fetal death. **a** entire-gestation exposure; **b** trimester-specific exposure. Dashed lines: estimates from individual double robust models by strata of gestational week of fetal death; Solid lines: point estimates from a nonlinear meta-regression; Ribbons: corresponding point-wise 95% confidence intervals

Considering the fine temporal resolution of the GA-specific outcomes, we further focused on the variation in effect of entire-gestation exposure to PM_2.5_ by gestational week of fetal death. We utilized a nonlinear meta-regression to derive GA-dependent effects. The results of the main model are shown in Fig. 3 and those from the other models (e.g., the unadjusted model) are shown in Fig. S2. The meta-regression of the DRMs showed a U-shaped pattern in the association between entire-gestation PM_2.5_ exposure and gestational week of fetal death (Fig. 3a). The smallest effect of PM_2.5_ exposure was observed for fetal deaths occurring around the 33^rd^ gestational week. A similar U-shaped pattern was observed the other two homogenous models (i.e., the PS model and covariate-adjusted models), which increased confidence in the findings.

We also estimated the GA-dependent effects for the three trimester-specific exposure periods (Fig. 3b). The GA- dependent effect of first-trimester PM_2.5_ exposure showed a U-shaped trend, similar to that for entire-gestation exposure (Fig. 3a). Although the GA- dependent effect of third-trimester PM_2.5_ exposure was only observed for fetal deaths occurring between the 37^th^ and 43^rd^ gestational week, we observed a comparable trend during over the entire gestational period (Fig. 3b). Therefore, the U-shaped effect might not be attributable to the effects of GA-specific exposure, but rather to differences in GA-specific outcomes, reflecting GA-related variations in the baseline risk of fetal death. The effect of second-trimester PM_2.5_ exposure generally decreased with GA, which might be explained by the increase in interval between exposure and outcome.

## Discussion

### Principal findings

By stratifying a birth and fetal death certificate dataset by GA (from 20 to 43 weeks) and analyzing GA-specific associations between PM_2.5_ and fetal death, we found that each 5-µg/m^3^ increment in PM_2.5_ exposure during the full gestation period significantly increased the odds of fetal death, by 8.1% (95% CI: 5.1–11.2%). Further, GA might modify the association between PM_2.5_ and fetal death. Among all GA-specific exposure-outcome pairs, that between PM_2.5_ exposure during early gestation (i.e., the first trimester) and early fetal death (i.e., 20–27 weeks) was the strongest. Our results suggest that early gestation is a critical time window for preventing the adverse effects of PM_2.5_ in terms of fetal death.

### Results in the context of what is known

To the best of our knowledge, this study is the first to comprehensively examine how GA modulates the effect of PM_2.5_ exposure on fetal death in the contiguous US. Although previous studies reported an association between prenatal PM_2.5_ exposure and fetal death or stillbirth, few focused on the modulating effect of GA [5, 13, 17–19]. Two studies have reported the effects of PM_2.5_ exposure on GA-specific outcomes. First, after examining data from 12 clinical centers across the US from 2002 to 2008, Mendola et al. divided stillbirths into four strata according to GA (23–26, 27–30, 31–36, and 37–42 weeks) and found that, for each IQR increase in average PM_2.5_ exposure during the entire pregnancy, the RRs were 1.12 (95% CI: 0.57–2.20), 1.53 (95% CI: 0.66–3.52), 0.71 (95% CI: 0.40–1.28), and 0.84 (95% CI: 0.44–1.59), respectively. Although the CIs were wide because of the small sample size in each stratum, the point estimates suggested that the adverse effects of PM_2.5_ on fetal survival were strongest at a GA ≤ 30 weeks [9]. Second, Ebisu et al. performed a matched case-control study in California from 2002 to 2009, and reported that each 7.23 µg/m^3^ increase in PM_2.5_ exposure during the whole gestation period increased the risk of stillbirth before and after 28 gestational weeks by 5% (95% CI: -1–12%) and 9% (95% CI: 1–17%), respectively [8]. Although these findings suggest that GA modulates the effect of PM_2.5_ exposure, the evidence is inconsistent. The heterogeneity in results between studies might be due to differences in their populations, design, exposure assessment accuracy, statistical models, or covariates, as well as pollution levels [13, 17, 18]. Additionally, although few studies have reported on the GA-specific effects of PM_2.5_ on fetal death, PM_2.5_ exposure during early gestation was found to have serious consequences, including small for GA [20], large for GA [20], infant mortality [21], reduced fetal growth [22], and preterm birth [23]. These findings are generally consistent with our study, which included a large sample size, simultaneously considered GA-specific exposure and outcomes, and improves our understanding of how GA moderates the effects of PM_2.5_ exposure.

### Clinical implications

Detecting critical exposure windows for ambient air pollution in terms of fetal death is important for elucidating the biological mechanisms of this phenomenon, as well as to optimize prenatal prevention and management strategies. Our results suggest that prevention of PM_2.5_ exposure during the first trimester should be a priority. The biological mechanisms underlying the association between PM_2.5_ and fetal death may include oxidative stress, systematic inflammation, endothelial function, and abnormal placental patterns [24, 25]. Early pregnancy might be the stage in which fetuses are most susceptible to PM_2.5_ exposure because this is when fetal implantation and placenta formation occur. Prenatal exposure to air pollutants in the first trimester might influence placental adaptation via changes in DNA methylation [26]. PM_2.5_ exposure during early gestation is likely to elevate the C-reactive protein concentration, which indicates an inflammatory response [27]. This systemic inflammation induced by PM_2.5_ might increase the risk of adverse birth outcomes, including fetal death.

### Different analysis models

Except for covariate-adjusted model, the current study utilized other different models to estimate the associations, including the propensity-score model and diagnostic-score model. The propensity-score method used exposure level to create pseudo-populations balanced for observed covariates. Because the propensity score was conducted without use of the outcome, this approach separated the design and analysis stages in an observation study, and was considered as a causal inference approach, which mimicked a randomized trial. However, propensity score would prioritize variables by their importance in predicting exposure received, not outcome. Variables that were related to the exposure but not to the outcome could undue influence in the propensity score, and increase the variance of the estimated exposure effect without decreasing bias [28]. Previous research suggested variables that were thought to be related to the outcome, regardless of whether they were related to the exposure should be included in a propensity score model [29]. Diagnostic-score model creates a pseudo-population makes the covariates comparable between different outcome groups (i.e., cases and controls), and this approach was regarded as a generalization of the matched case-control design. Unlike the propensity score model, diagnostic-score model focused on achieving balance on the variables highly predictive of the outcome. The variables most predictive of the outcome were typically of utmost concern, as such variables might cause the most bias if left unbalanced [30]. The double robust model combined the propensity score and covariate adjustment approaches, so that correctly specifying the presumption for either approach can make sure the estimates as unbiased. The heterogeneity in GA-specific estimates might derive from unmeasured confounders. In other words, the heterogeneity should be minimized if confounders were well controlled in a model. Accordingly, in our meta-analysis results, covariate-adjusted model, propensity-score model, and double robust model reported GA-specific estimates with less heterogeneity. The heterogeneity among different models might arise from the covariate selection, unmeasured confounders, and models hypotheses, therefore it was essential to conduct additional exploration through theoretical analyses, such as statistical simulations.

### Strengths and limitations

Compared to our previous study, the current findings are novel in the following ways: first, we confirmed a direct effect of PM_2.5_ exposure on fetal death, i.e., independent of GA. In our previous study, we proposed an indirect pathological pathway (i.e., PM_2.5_ → short GA → immaturity → fetal death) to explain why PM_2.5_ exposure increases the risk of fetal death. To evaluate the overall effect of PM_2.5_ exposure, GA was not adjusted in the models and the OR of fetal death was estimated to be 1.47 (95% CI: 1.28–1.61) for each 5-µg/m^3^ increase in prenatal PM_2.5_ exposure [31]. Stratifying the data by GA in the current study enabled us to control for the mediating effect thereof. The significant results indicate a direct effect of PM_2.5_ exposure on fetal death. Second, we focused on the mechanism through which GA could modify the effect of PM_2.5_ exposure on fetal death. We generated new data by analyzing GA-specific exposure-outcome pairs. This was a unique approach; previous studies mostly distinguished only between trimester-specific exposures. We found that the adverse effects on PM_2.5_ with respect to fetal death differed by GA. Further, the association between PM_2.5_ exposure and early fetal death was strongest during the first trimester. These results suggest that GA modifies the effect of PM_2.5_ exposure in a complex way, and that more attention should be paid to PM_2.5_ exposure during early pregnancy.

Our study had several limitations. First, exposure misclassification is possible; although we matched the exposure data according to the county of maternal residence at delivery, we did not consider variations in exposure within counties. However, due to the smaller within-county variation of PM_2.5_ compared to the between-county variation, the model has successfully captured the majority of exposure variation.Furthermore, we did not account for indoor air pollution or long-distance migration during pregnancy. However, we assume that pregnant women are generally unlikely to migrate. Moreover, the high temporal correlation of PM_2.5_ observed between distant locations has contributed to mitigating the potential impact of misclassification to a considerable degree. Additionally, we only had access to monthly PM_2.5_ concentration data, so inaccuracies in the assessments of trimester exposure are possible; other maternal and fetal datasets with more refined exposure data (weekly or daily concentrations) are needed to further investigate this topic. Second, residual confounding bias is possible, such as lacking of socio-economic indicators and lifestyle data. The models included the less detailed binary variables (yes or no) for maternal smoking and maternal alcohol use, given the relatively low occurrences of maternal smoking (9.24%) and maternal alcohol consumption (1.21%), the dichotomous variates could well-control these two confounders. Notably, other information of physical activity, dietary habit, drug use and mental health was missing. The dataset also lacked corresponding information regarding the father, which could have served as a proxy for assessing the socio-economic status of the family. We introduced the dummy variables of residential state and year of birth, season of birth to control for unmeasured spatiotemporal variation, which to some extent controlled the confounding bias. However, the relationship between some covariates and fetal death might be in complex nonlinear patterns. Therefore, a few risks could be unmeasured due to the less specific covariates. Also, the causal inference models (i.e., propensity score model) was utilized based on the assumption that there was no unmeasured confounder, therefore the results should be interpreted carefully. Third, the differences in results among the three exposure periods should be interpreted carefully. For instance, the GA-dependent effect of first-trimester PM_2.5_ exposure was similar to that of full gestation exposure (Fig. 3b), which might be partly due to the similarity in sample size. The effect of first-trimester exposure to averaged levels of PM_2.5_ was estimated for all samples, because all fetal deaths occurred after the 20^th^ gestational week, i.e. after first-trimester exposure. In contrast, second-trimester PM_2.5_ exposure did not affect early fetal death, while third-trimester PM_2.5_ exposure affected neither early nor late fetal death. To fully understand the modulating effect of GA on the relationship between PM_2.5_ exposure and outcomes, an advanced statistical model is needed. Fourth, the sample population was selected from1989 to 2004 due to the accessibility of the residential locations, and the poor timeliness of dataset limited the generalizability of the results to contemporaneous settings. Therefore, our findings should be confirmed by future studies using more timely datasets.

## Conclusion

Registration data for 47.8 million fetuses in the contiguous US were analyzed in this population study, making it the largest study on the association between fetal death and prenatal PM_2.5_ exposure to take GA into account. Overall, a 5-µg/m^3^ increase in prenatal exposure to PM_2.5_ was associated with a 8.1% (95% CI: 5.1–11.2%) increase in the odds of fetal death in the US. The effect of GA on outcomes was U-shaped, i.e., there were stronger effects for early and late GAs. These results provide additional evidence of the adverse effects of PM_2.5_ exposure. Additionally, we identified acute effects of PM_2.5_ exposure, and a long-term effect of first-trimester exposure. Improvements in air quality and other protective measures, especially during the first trimester, could help prevent fetal deaths.

### Supplementary Information

**Additional file 1:** **Table S1.** Characteristics of study participants (from gestational 20^th^ week to 43^rd^ week). **Figure S1.** Results of meta-analysis of the association between fetal death and 5 µg/m^3^ increment in gestational PM_2.5_ exposure stratified by gestational week. The pooled odds ratios (ORs) and their corresponding 95% confidence intervals (CIs) were derived from meta-analysis with random effects. Heterogeneity among individual estimates stratified by gestational week was evaluated using I^2^. **Figure S2.** Association between fetal death and gestational exposure to PM_2.5_ stratified by the GA (from 20^th^ week to 43^rd^ week) and estimated using different models (the green ribbon represents the 95% confidence intervals of the double robust model).

## Data Availability

The data underlying this article are based on publicly available datasets, and data sources have been provided in the article.
