# Supplementary material for: Gestational age modifies the association between exposure to fine particles and fetal death: findings from a nationwide epidemiological study in the contiguous United States
Source: Environ Health. 2023 Sep 14;22:65. doi: 10.1186/s12940-023-01016-4 (PMC10500914; doi:10.1186/s12940-023-01016-4)
Supplement: Supplementary file 1 — Additional file 1: Table S1. Characteristics of study participants (from gestational 20th week to 43rd week). Figure S1. Results of meta-analysis of the association between fetal death and 5 µg/m3 increment in gestational PM2.5 exposure stratified by gestational week. The pooled odds ratios (ORs) and their corresponding 95% confidence intervals (CIs) were derived from meta-analysis with random effects. Heterogeneity among individual estimates stratified by gestational week was evaluated using I2. Figure S2. Association between fetal death and gestational exposure to PM2.5 stratified by the GA (from 20th week to 43rd week) and estimated using different models (the green ribbon represents the 95% confidence intervals of the double robust model). [file 12940_2023_1016_MOESM1_ESM.docx]

**Supplemental materials**

**Gestational age modifies the association between exposure to fine particles and fetal death: Findings from a nationwide epidemiological study in the contiguous United States**

Mingkun Tong^1^, Weiwei Lin^2^, Hengyi Liu^1^, Jicheng Gong^3^, Junfeng (Jim) Zhang^4,5^, Tao Xue^1,3,6,*^

^1^ Institute of Reproductive and Child Health, National Health Commission Key Laboratory of Reproductive Health and Department of Epidemiology and Biostatistics, Ministry of Education Key Laboratory of Epidemiology of Major Diseases (PKU), School of Public Health, Peking University Health Science Centre, Beijing, China

^2^ Department of Occupational and Environmental Health, School of Public Health, Sun Yat-Sen University, Guangzhou, China

^3^ State Environmental Protection Key Laboratory of Atmospheric Exposure and Health Risk Management and Center for Environment and Health, Peking University, Beijing, China

^4^ Global Health Research Center, Duke Kunshan University, Kunshan, China.

^5^ Nicholas School of the Environment and Duke Global Health Institute, Duke University, Durham, USA.

^6^ Advanced Institute of Information Technology, Peking University, Hangzhou, Zhejiang, China

Table S1 Characteristics of study participants (from gestational 20^th^ week to 43^rd^ week).

| Characteristics | Subgroup | N (%) |
| --- | --- | --- |
| Season | Dec-Jan-Feb | 11,553,729 (24.15) |
|  | May-Apr-Mar | 11,734,612 (24.53) |
|  | Jun-Jul-Aug | 12,230,838 (25.56) |
|  | Sep-Oct-Nov | 12,326,265 (25.76) |
| Year | 1989 | 744,068 (1.56) |
|  | 1990 | 3,172,239 (6.63) |
|  | 1991 | 3,142,678 (6.57) |
|  | 1992 | 3,119,445 (6.52) |
|  | 1993 | 3,084,756 (6.45) |
|  | 1994 | 3,114,862 (6.51) |
|  | 1995 | 3,072,623 (6.42) |
|  | 1996 | 3,058,404 (6.39) |
|  | 1997 | 3,055,628 (6.39) |
|  | 1998 | 3,104,986 (6.49) |
|  | 1999 | 3,120,424 (6.52) |
|  | 2000 | 3,206,245 (6.70) |
|  | 2001 | 3,190,819 (6.67) |
|  | 2002 | 3,189,376 (6.67) |
|  | 2003 | 3,224,635 (6.74) |
|  | 2004 | 3,244,256 (6.78) |
| Gestational age (week) | 20 | 41,859 (0.09) |
|  | 21 | 51,958 (0.11) |
|  | 22 | 58,261 (0.12) |
|  | 23 | 59,863 (0.13) |
|  | 24 | 66,489 (0.14) |
|  | 25 | 72,493 (0.15) |
|  | 26 | 83,453 (0.17) |
|  | 27 | 88,639 (0.19) |
|  | 28 | 115,489 (0.24) |
|  | 29 | 136,607 (0.29) |
|  | 30 | 186,983 (0.39) |
|  | 31 | 234,411 (0.49) |
|  | 32 | 320,292 (0.67) |
|  | 33 | 459,253 (0.96) |
|  | 34 | 761,956 (1.59) |
|  | 35 | 1,204,897 (2.52) |
|  | 36 | 2,010,709 (4.20) |
|  | 37 | 3,763,474 (7.87) |
|  | 38 | 7,552,112 (15.78) |
|  | 39 | 11,420,439 (23.87) |
|  | 40 | 10,518,037 (21.98) |
|  | 41 | 5,710,721 (11.94) |
|  | 42 | 2,002,070 (4.18) |
|  | 43 | 924,979 (1.93) |
| Maternal age (years) | <15 | 380,848 (0.80) |
|  | 16~20 | 6,620,435 (13.84) |
|  | 21~25 | 10,963,947 (22.92) |
|  | 26~30 | 12,762,746 (26.67) |
|  | 31~35 | 9,758,869 (20.40) |
|  | 35~40 | 3,781,213 (7.90) |
|  | 41~45 | 548,850 (1.15) |
|  | ≥46 | 18,849 (0.04) |
|  | Missing | 3,009,687 (6.29) |
| Weight gain during pregnancy (pound) | <15 | 3,886,940 (8.12) |
|  | 16~20 | 3,898,454 (8.15) |
|  | 21~25 | 5,186,429 (10.84) |
|  | 26~30 | 6,856,318 (14.33) |
|  | 31~35 | 5,158,879 (10.78) |
|  | 36~41 | 4,612,067 (9.64) |
|  | 41~45 | 2,384,029 (4.98) |
|  | ≥46 | 4,069,096 (8.50) |
|  | Missing | 11,793,232 (24.65) |
| History of other termination | No | 35,668,116 (74.55) |
|  | Once | 7,964,854 (16.65) |
|  | Twice | 2,615,786 (5.47) |
|  | Thrice and more | 1,339,580 (2.80) |
|  | Missing | 257,108 (0.54) |
| Marital status | Yes | 32,572,085 (68.08) |
|  | No | 15,146,826 (31.66) |
|  | Missing | 126,533 (0.26) |
| Has born at least a livebirth | Yes | 28,226,251 (58.99) |
|  | No | 19,380,070 (40.51) |
|  | Missing | 239,123 (0.50) |
| Prenatal care attendance | Yes | 45,579,524 (95.26) |
|  | No | 639,584 (1.34) |
|  | Missing | 1,626,336 (3.40) |
| Plurality | 1 | 46,348,098 (96.87) |
|  | 2 | 1,410,287 (2.95) |
|  | 3 + | 85,177 (0.18) |
|  | Missing | 1,882 (0.00) |
| Born in hospital | Yes | 47,421,693 (99.11) |
|  | No | 423,549 (0.89) |
|  | Missing | 202 (0.00) |
| Maternal tobacco usage | No | 31,950,491 (66.78) |
|  | Yes | 4,420,984 (9.24) |
|  | Missing | 11,473,969 (23.98) |
| Maternal alcohol usage | No | 36,925,200 (77.18) |
|  | Yes | 580,957 (1.21) |
|  | Missing | 10,339,287 (21.61) |
| Chronic diabetes | Yes | 1,330,326 (2.78) |
|  | No | 45,637,482 (95.39) |
|  | Missing | 877,636 (1.83) |
| History of hypertension | Yes | 348,646 (0.73) |
|  | No | 46,619,162 (97.44) |
|  | Missing | 877,636 (1.83) |
| Sex | Male | 24,510,455 (51.23) |
|  | Female | 23,334,989 (48.77) |
|  | Missing | 0 (0.00) |
| Mother's ethnicity | White | 36,708,200 (76.72) |
|  | Africa American | 7,798,101 (16.30) |
|  | Chinese | 388,106 (0.81) |
|  | American Indian / Alaskan Native | 278,915 (0.58) |
|  | Japanese | 90,639 (0.19) |
|  | Hawaiian | 17,784 (0.04) |
|  | Others | 2,563,699 (5.36) |
| Father's ethnicity | White | 32,783,014 (68.52) |
|  | Africa American | 5,306,181 (11.09) |
|  | Chinese | 348,742 (0.73) |
|  | American Indian / Alaskan Native | 206,914 (0.43) |
|  | Japanese | 68,696 (0.14) |
|  | Hawaiian | 15,861 (0.03) |
|  | Others | 9,116,036 (19.05) |


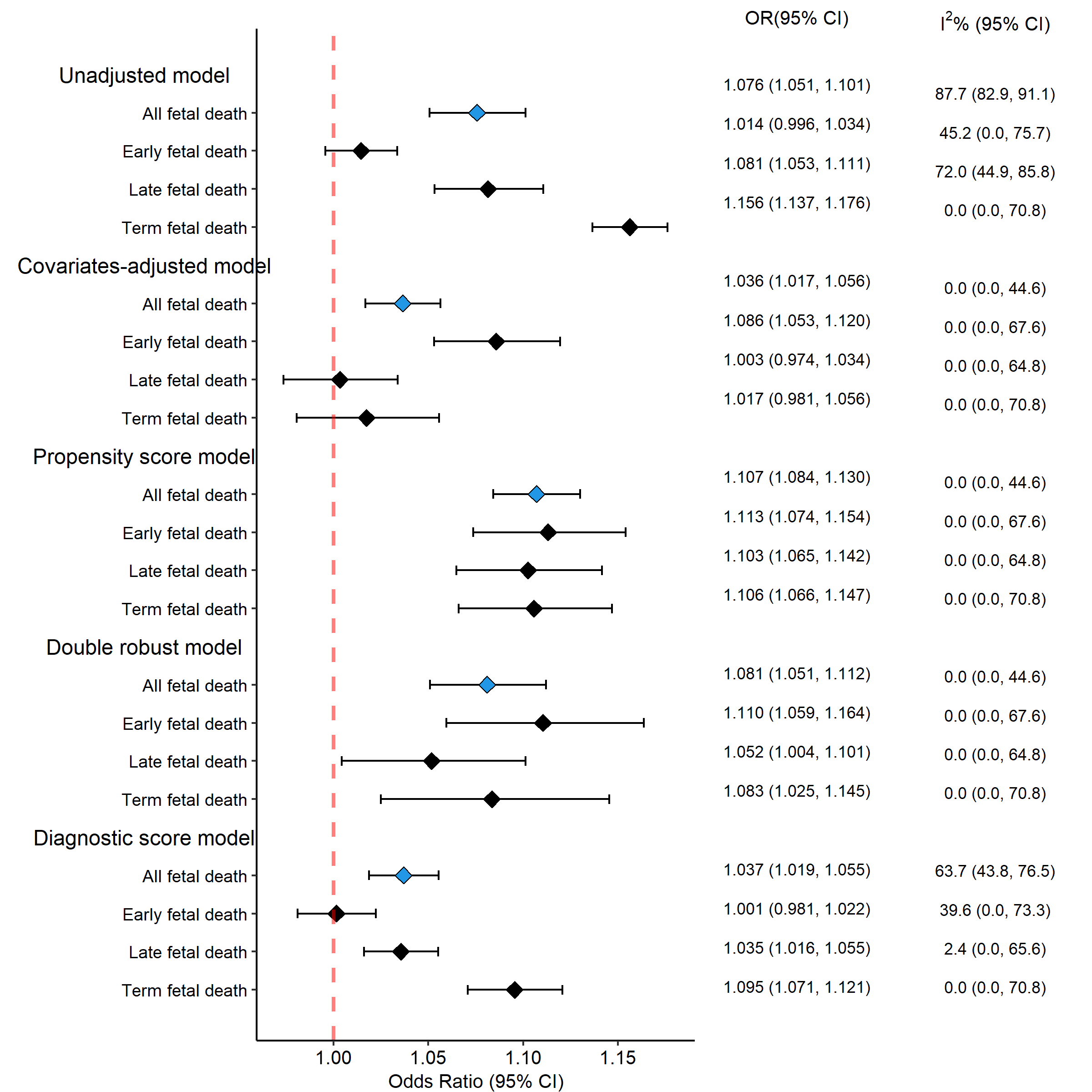


Figure S1 Results of meta-analysis of the association between fetal death and 5 µg/m^3^ increment in gestational PM_2.5_ exposure stratified by gestational week. The pooled odds ratios (ORs) and their corresponding 95% confidence intervals (CIs) were derived from meta-analysis with random effects. Heterogeneity among individual estimates stratified by gestational week was evaluated using I^2^.


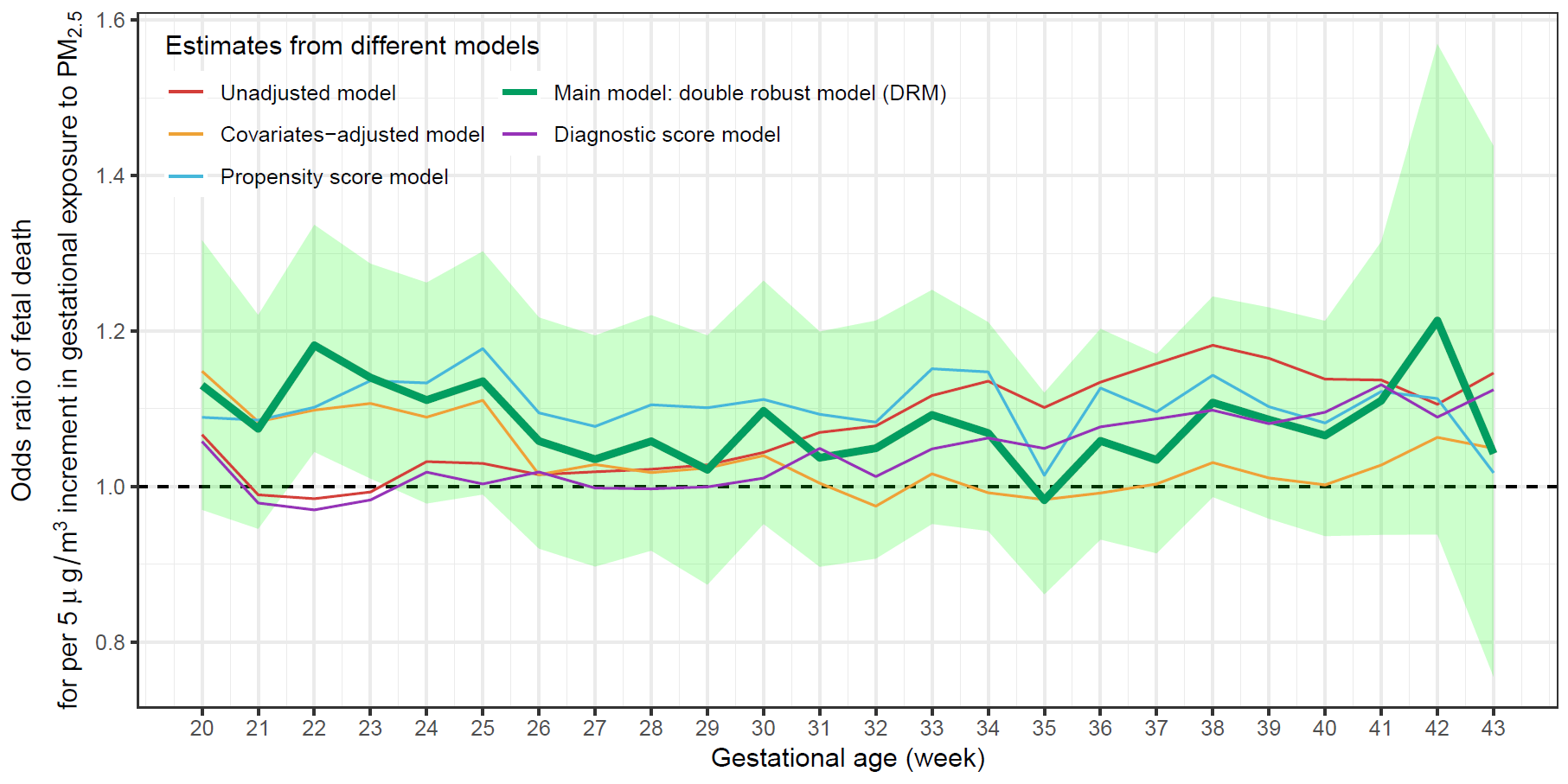


Figure S2 Association between fetal death and gestational exposure to PM_2.5_ stratified by the GA (from 20^th^ week to 43^rd^ week) and estimated using different models (the green ribbon represents the 95% confidence intervals of the double robust model).
